# The role of algae, fungi, and insect-derived proteins and bioactive peptides in preventive and clinical nutrition

**DOI:** 10.3389/fnut.2024.1461621

**Published:** 2024-10-10

**Authors:** Mohammed Ahmed Yimam, Martina Andreini, Sara Carnevale, Maurizio Muscaritoli

**Affiliations:** ^1^Department of Science, Technology and Society, University School for Advanced Studies IUSS Pavia, Pavia, Italy; ^2^Department of Translational and Precision Medicine, Sapienza University of Rome, Rome, Italy; ^3^Department of Public Health, College of Health Science, Woldia University, Woldia, Ethiopia; ^4^Belcolle Hospital, Viterbo, Italy

**Keywords:** algae, fungi, insect, protein, peptides, cancer, cardiovascular disease, dementia

## Abstract

The current global trend in the nutrition, epidemiologic and demographic transitions collectively alarms the need to pursue a sustainable protein diet that respects ecosystem and biodiversity from alternative sources, such as algae, fungi and edible insects. Then, changing the nutrition reality is extremely important to impede the global syndemic of obesity, undernutrition and climate change. This review aims to synthesize the published literature on the potential roles of alternative proteins and their derived bioactive peptides in preventive and clinical nutrition, identify research gaps and inform future research areas. Google Scholar and PubMed databases from their inception up to 30 June 2024 were searched using keywords to access pertinent articles published in English language for the review. Overall, proteins derived from algae, fungi, and edible insects are high-quality proteins as animal sources and demonstrate significant potential as a sustainable source of bioactive peptides, which are metabolically potent and have negligible adverse effects. They show promise to prevent and treat diseases associated with oxidative stress, obesity, diabetes, cancer, cardiovascular disease (especially hypertension), and neurodegenerative diseases. Given the abundance of algae, fungi and insect peptides performed *in vitro* or *in vivo* animals, further clinical studies are needed to fully establish their safety, efficacy and practical application in preventive and clinical nutrition. Additionally, social and behavioral change communication strategies would be important to increase health awareness of nutritional benefits and promote consumer acceptance of alternative protein sources.

## 1 Introduction

Nutrition is a crucial pillar of human life, health and development (1, 2). Good nutrition ensures health and wellness at each stage of the human life cycle, such as pregnancy, infancy, childhood, adolescence, adulthood, and older age (3). Unfortunately, the double burden of malnutrition, i.e., concurrent manifestation of both undernutrition and overnutrition (overweight and obesity), may become manifest within the life course (4, 5). Although the burden of malnutrition mainly affects low and middle-income countries, countries passing the economic transitions also face the problem (6). Alongside, shifting of the overall dietary structure over time, i.e., the trend of the current nutrition transition, is associated with unrelenting non-communicable diseases (NCDs). Therefore, changing the nutrition reality is extremely important because a low-quality diet ignites a double burden of malnutrition (5, 7, 8).

By the year 2050, the world population is projected to be nearly 10 billion (9), the food demand is expected to increase by 56% (10), and animal-derived protein demand will be twofold (11), requiring a sustainable food system (12). Of significant concern, the increased consumption of red and processed meat globally contributes to an abrupt increase in the attributable burden of diet-related NCDs, including colorectal cancer, diabetes, and coronary heart disease, which are markedly risen in Northern and Eastern European countries as well as island countries in the Caribbean and Oceania (13). The demand and consumption of meat is also rapidly increasing in developing countries due to urbanization and rapid income growth (14).

**Figure 1:**
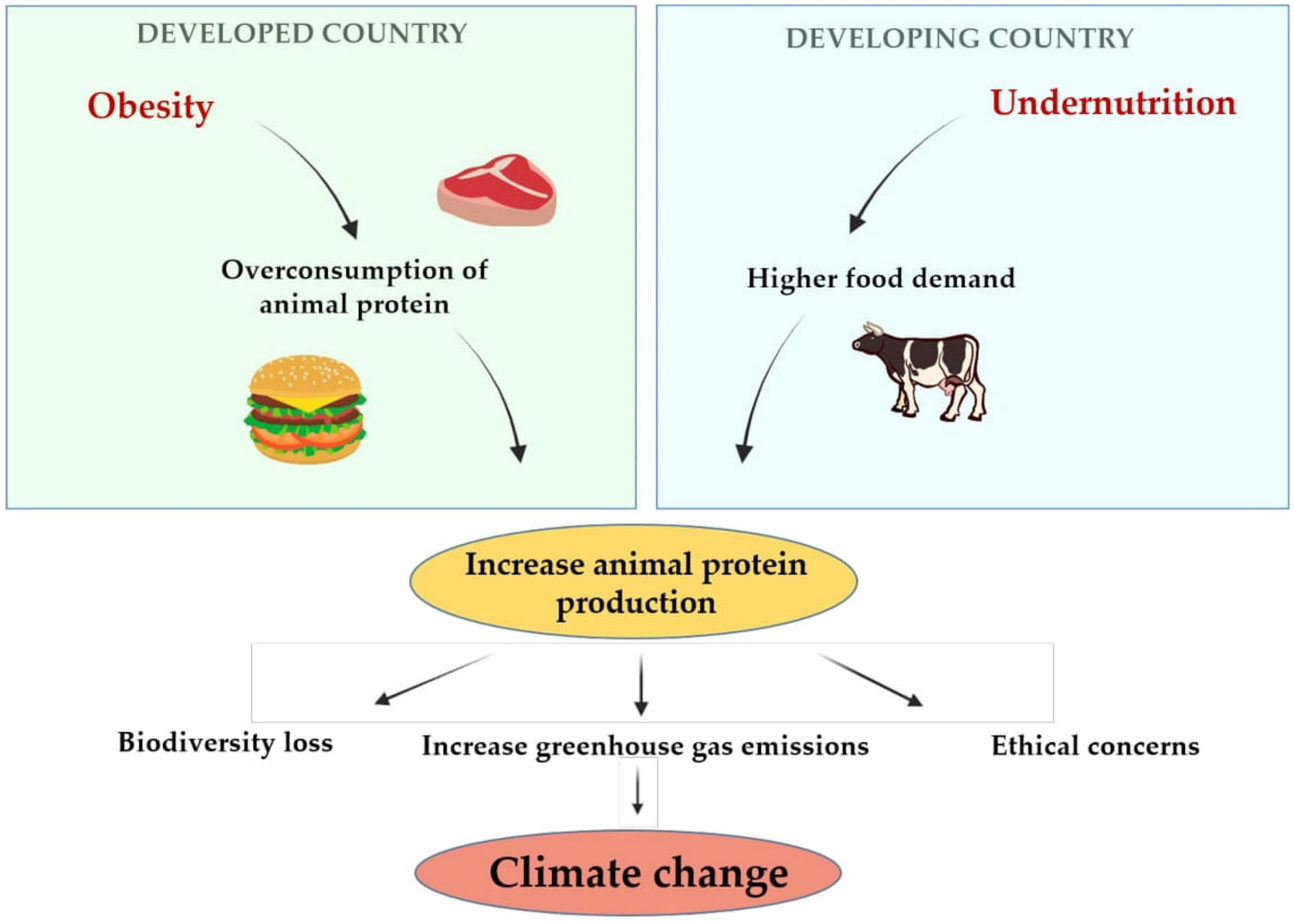
The relationship between obesity, undernutrition and climate change.

Furthermore, raising livestock and animal meat consumption is associated with higher greenhouse gas emissions (15, 16), higher consumption of water and land use (17), human-induced terrestrial biodiversity loss (18, 19), contracting zoonosis and antibiotic resistance (20), and raised ethical concerns about animal welfare (21). These impacts clearly illustrate the need to pursue a sustainable healthy diet from alternative protein sources, such as algae, fungi, and edible insects, to achieve global food security without expanding crop or pastureland, deprived of increasing greenhouse gas emissions and without devaluing health (11, 17, 22–26). Figure 1 summarizes the relationship between obesity, undernutrition and climate change.

Shifting towards alternative protein sources is vital to meet most of the United Nations’ sustainable development goals and to achieve a net-zero greenhouse gas emission target indicated in the Paris Agreement (27). In accordance with this, in 2022, the sixth international panel on climate change urged governments to prioritize sustainable healthy diets to feed the projected population by 2050, while mitigating the effects of climate change (28). The environmental and nutritional advantage inspired the concept of a sustainable diet, i.e., a diet that respects biodiversity and ecosystems, that is nutritionally adequate, safe, healthy and at the same time culturally acceptable, and affordable, as highlighted by the Food and Agriculture Organization of the United Nations (29, 30). The role of a sustainable diet from plant origin on metabolic syndrome in humans is extensively explored (31–34). Emerging evidence also spotlights the potential role of alternative proteins (algae, fungi and insects) and their derived bioactive peptides, i.e., a group of biological molecules activated by extraction from parental proteins, to offer therapeutic advantages by modulating metabolic pathways (35). Particularly, bioactive peptides are known for their safety, tolerability, and minimal risk of adverse effects (36). The quests of these proteins and derived peptides in the context of preventive and clinical nutrition present a promising road to uncover innovative dietary strategies and therapeutic approaches. Given no previous review in the area, this narrative review aims to (1): explore the current research on the potential roles of alternative proteins and their derived bioactive peptides in preventive and clinical nutrition (2); identify gaps in the literature; and (3) inform future research.

## 2 Methods

Google Scholar and PubMed databases from their inception up to 30 June 2024 were searched using keywords to access pertinent articles published in English-language for the review. The reference lists of included studies were additionally screened to locate further relevant literature. We used the following keywords for the review: “algae OR algal protein OR algae peptide + Y,” “fungi OR fungal protein OR fungi peptide + Y,” “Insect OR insect protein OR insect peptide +Y,” where Y indicates hypertension, obesity, T2DM, dementia, cancer, and sarcopenia.

### 2.1 The role of algae-derived protein and bioactive peptides in preventive and clinical nutrition

**Figure 2:**
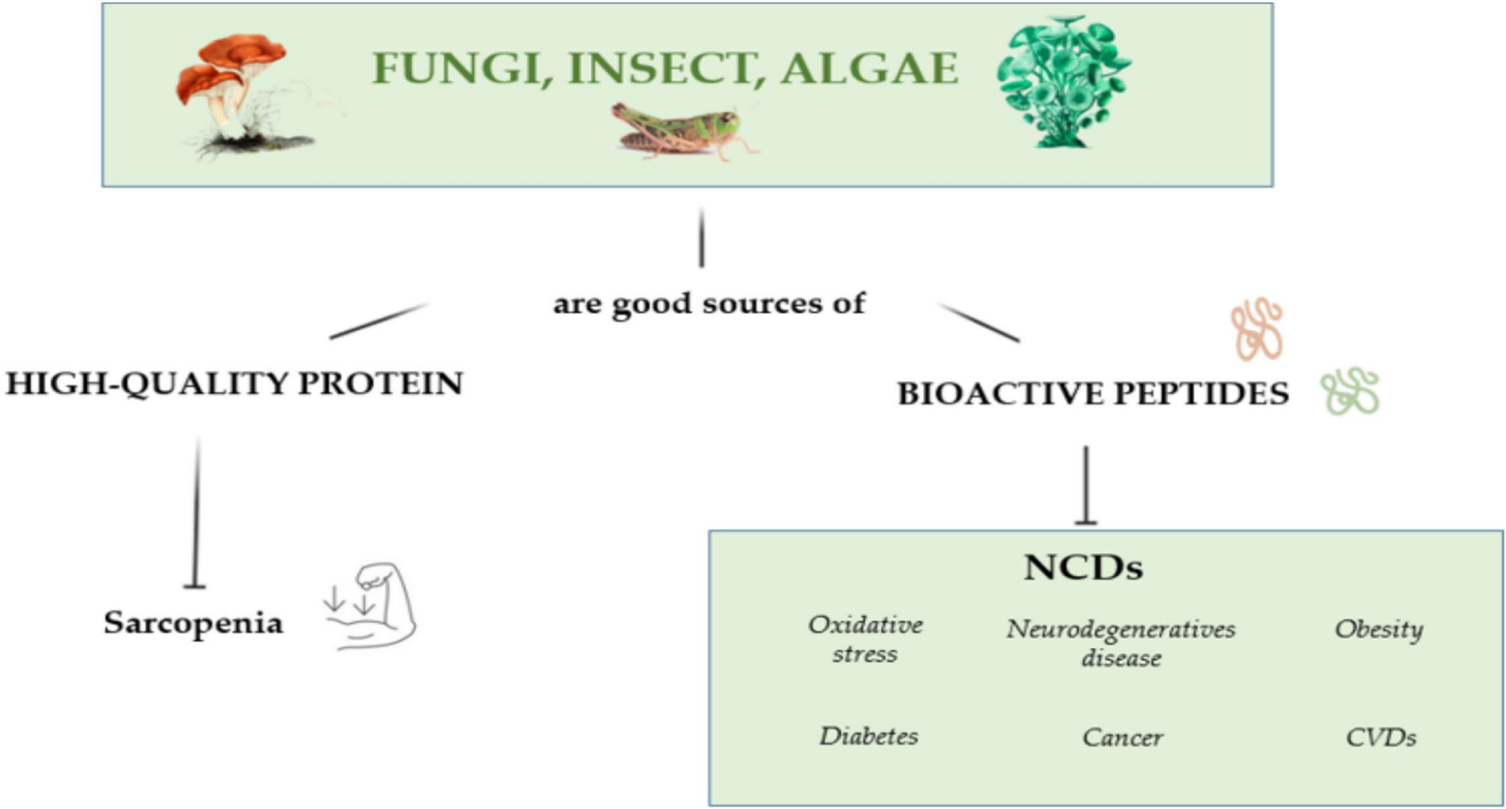
The possible positive effects of fungi, insects and algae in preventive and clinical nutrition.

Algae are protein-rich marine resources that have a rapid growth rate, can adapt to extreme and competitive environments, and contain numerous health-promoting compounds (37) and proteins, such as lectins, phycobiliproteins, mycosporine-like amino acids, derived hydrolysates, and bioactive peptides (38). Commercially available microalgae, such as spirulina (*Cyanobacterium Arthrospira platensis*) and *Chlorella vulgaris* contain up to 68% protein by dry weight (39, 40), whereas red algae contain up to 47% of protein (41). Proteins extracted from spirulina and chlorella have a high degree of *in vitro* digestibility and contain all essential amino acids (42, 43). Further studies assessing the *in vivo* digestibility of these proteins are necessary. Additionally, algae-derived bioactive peptides exhibited anti-microbial, anti-mutagenic, anti-inflammatory, anti-diabetic, anti-hypertensive, anti-oxidant, anti-tumor, neurophysiological and hepatoprotective activity (44–47). Figure 2 summarizes the role of algae, fungi and insect derived bioactive peptides in preventive and clinical nutrition.

#### 2.1.1 Anti-sarcopenic effect of algae-derived protein and bioactive peptides

By the year 2050, the number of individuals aged 60 years and above will double (2.1 billion), particularly those over 85 years will triple (426 million) (48). Sarcopenia, the key hallmark of aging, refers to the progressive loss of muscle mass and loss of strength or performance (49). Sarcopenia negatively affects the quality of life of the individual (50, 51), burdens the public health sector (52, 53) and increases mortality rates (54, 55). Daily intake of adequate (30 g/meal) high biological value, leucine-rich proteins with physical activity is crucial to counteract sarcopenia by maximizing muscle protein synthesis (MPS) rate (56).

Food protein and their derived bioactive peptides could be a promising option in this regard (57, 58). For example, *Spirulina platensis*, is a potential protein supplement. A preceding study was conducted to examine the anti-sarcopenic effect of Spirulina protein hydrolysate (SPH) in dexamethasone-treated C_2_C_12_ cells. The results indicated that SPH inhibits muscle atrophy mainly by activating the Akt/FoxO3a signaling pathway, specifically by increasing MyoD1, Myf5, and myogenin and decreasing Atrogin-1, MuRF-1, and FoxO3a (59). However, further studies on algae-derived peptides using animal models is required to affirm SPH as a solution for the prevention, and treatment of sarcopenia. Recently, randomized controlled trials performed among young adults (age: 22 ± 3 years) have shown that algae-derived protein (spirulina, and chlorella) ingestion stimulates muscle protein synthesis similar to mycoprotein (60). However, further research in older adults is warranted to translate into the prevention and treatment of sarcopenia.

#### 2.1.2 Anti-obesity effect of algae-derived protein and bioactive peptides

Globally, obesity is a major public health problem primarily associated with the consumption of high-fat diets and ultra-processed foods (7, 61). It is attributable to the global incidence of cardiovascular disease, cancer, type 2 diabetes mellitus, osteoarthritis, work disability, and sleep apnea (62). As a consequence, algae-derived proteins and bioactive peptides could serve as anti-obesity agents (63, 64). In high-fat diet-fed mice, the anti-obesity effects of Spirulina-derived protein (SPP) or peptide (SPPH) are higher to the whole Spirulina (WSP), SPP is slightly lower than SPPH, under the same dose (2 g/kg per day). Overall, SPPH showed good anti-obesity effects, such as reduction of body weight (39.8% ± 9.7%), lowering serum glucose (23.8% ± 1.6%), decreasing total cholesterol (20.8% ± 1.4%), while positive drug Simvastatin (10 mg/kg per day) had the corresponding values: 8.3 ± 4.6, 24.8 ± 1.9% and − 2.1% ± 0.2%, respectively. SPPH anti-obesity effects modulate the expressions of key genes in the brain (Acadm, Gcg) and liver (Retn, Fabp4, Ppard, and Slc27a1), which are linked with lipid metabolism and accumulation (65). A peptide, CANPHELPNK, identified from enzymatic hydrolysates of *Spirulina platensis* protein showed the best anti-proliferative activity on preadipocytes 3 T3-L1 (60.08%), which was close to that of Simvastatin (70.32%), at 2 mg/ml. Furthermore, the two peptides NPVWKRK and CANPHELPNK were also demonstrated to significantly reduce the accumulation of triglyceride at 600 μg/ml, with 23.7 and 19.5%, respectively compared with control (66). Moreover, in high fat diet-fed rats, supplementation with *Chlorella vulgaris* effectively reduced total serum lipids, liver triglycerides, and cholesterol (67).

Intriguingly, a systematic review and meta-analysis of randomized controlled trials in humans showed that spirulina supplementation significantly reduces body weight, body fat percentage, and waist circumference (68). It also decreases triglycerides and total cholesterol levels in patients with type 2 diabetes, metabolic syndrome, overweight, or obesity (69). Spirulina is “generally recognized as safe” for human consumption (70). Nevertheless, rare potential adverse effects, such as acute rhabdomyolysis (71) and anaphylaxis (72) were reported. Therefore, an allergy risk assessment before supplementation is strongly suggested.

#### 2.1.3 Anti-hypertensive effect of algae-derived proteins and bioactive peptides

Hypertension shares a large quota as a risk factor for CVD (73). It is further leads to end-stage renal disease, stroke, disability, dementia and mortality (74–76). To lessen hypertension, angiotensin-converting enzyme (ACE) inhibition using synthetic drugs is the basic step, despite undesirable side effects, including dry cough, angioedema, disturbance, and skin rash are associated with the drug (77). Therefore, pursuing anti-hypertensive bioactive peptides from natural food sources, such as algae has paramount importance (78).

*In silico* and *in vitro* assessment showed that bioactive peptides (Alcalase, bromelain, papain, pepsin) of *A. platensis* demonstrated ACE inhibitory activity (80% inhibition rate at a level of 1.0 mg/ml) (79). Moreover, oral administration of protein hydrolysate from *Chlorella Vulgaris* (5 mg/kg of body weight) to spontaneously hypertensive rats (SHR) effectively reduced the systolic blood pressure by 50 mmHg, verifying the potent antihypertensive effects of certain peptides, equivalent to synthetic drugs (80). Interestingly, a systematic review and meta-analysis of randomized controlled trials in humans showed that chlorella supplementation (4 g/day) for 8 weeks and more, significantly reduced total cholesterol, low-density lipoprotein, systolic and diastolic blood pressure in hypertensive patients (81).

#### 2.1.4 Anti-diabetic effect of algae-derived protein and bioactive peptides

Type 2 diabetes (T2DM) is a metabolic disorder that accounts for more than 90% of patients with diabetes, which leads to microvascular (neuropathy, retinopathy and nephropathy) and macrovascular (CVD) complications. In addition, T2DM has a significant impact on patients’ lives and puts a huge burden on healthcare systems (82, 83). The existing anti-diabetic agents are often associated with side effects (84); as a result, there is a growing interest in the use of food protein/peptides as a potential remedy for the prevention and management of T2DM (85), especially in its early forms.

Bioactive peptides derived from various algae species, such as *A. platensis* (79, 86, 87), *Chlorella* sp. (88), and *P. palmata* (89), have demonstrated anti-diabetic bioactivities via inhibiting Dipeptidyl peptidase IV (DPP-IV), and carbohydrate-digesting enzyme inhibition, i.e., α-amylase and α-glucosidase, thereby reduced the postprandial blood glucose absorption. Literature has shown that *Spirulina platensis*-derived anti-hyperglycemic peptide exhibited the best inhibition on α-amylase (62%), α-glucosidase (90%) and DPP-IV (49%) (87). Thus, the inhibitory activity of α-glucosidase was remarkably higher than α-amylase (87). This could be useful to decrease side effects due to the abnormal bacterial fermentation of undigested saccharides in the colon, which commonly occurs following excessive inhibition by α-amylase (90).

In streptozotocin-induced diabetic mice (SIDM), oral administration of *P. palmata* crude protein hydrolysate influenced the incretin system by directly upregulating the secretion of glucagon-like peptide 1 (GLP-1) and glucose-dependent insulinotropic polypeptide (GIP) (89).

#### 2.1.5 Anti-dementia role of algal proteins

Among individuals diagnosed with dementia, a neurodegenerative disease, such as Alzheimer’s disease (AD) shares a large part (60%), which arises when nerve cells in the central nervous system gradually lose function due to proinflammatory stimulus that facilitates the generation of neurotoxicity substances, and eventually die (91, 92). Neurodegenerative disease affects the patient’s quality of life (93), increases the burden for caregivers (94) and poses a higher economic cost (95). Though the available treatments may relieve symptoms, no known curative treatment is found for neurodegenerative diseases. As a consequence, food-derived peptides could have the potential to mitigate neurological inflammation, thereby improving cognitive performance (91, 96). A previous study indicated that dietary administration of 1 and 2% *Spirulina platensis* for 16 weeks in high fat diet fed mice significantly improved spatial learning and memory performance via inhibiting Aβ accumulation, tau-hyperphosphorylation, and neuroinflammation in the hippocampus (97). This study provides further evidence for the application of *Spirulina platensis* derived protein as a functional supplement for the treatment of AD. In support, the effects of spirulina intake (500 mg/day spirulina powder versus placebo twice a day for 12 weeks) among 60 patients with AD significantly improved their cognitive function, as evidenced by the mini-mental state examination score (MMSE). Additionally, spirulina intake decreased high-sensitivity C-reactive protein, fasting glucose, insulin resistance, and increased insulin sensitivity compared with the placebo (98).

#### 2.1.6 Anti-cancer role of algae-derived protein and bioactive peptides

Cancer is the leading cause of death next to CVD (99). Cancer metastasis to vital organs of the body usually results in massive tissue destruction, loss of life-sustaining functions, and death (100). The investigation of anticancer drugs derived from natural products has become a hot topic in the field of cancer research because of their lower economic cost and fewer side effects (101, 102).

Dietary algae such as *Chlorella vulgaris* (103), *Chlorella pyrenoidosa* (104), *Spirulina platensis* (105), and *Enteromorpha prolifera* (106) have shown significant anticancer bioactivities. Literature has shown that enzymatic hydrolysates of *A. platensis* protein at a concentration of 0.5 mg/ml exhibited comparable inhibitory effects against breast cancer cells (MCF-7), lung cancer cells (A549), gastric cancer cells (SGC-7901), colon cancer cells (HT-29), and liver cancer cells (HepG2) compared to the positive control drug 5-flurouracil (5-FU) (105, 107, 108). Moreover, the protein-derived peptide from *Chlorella vulgaris* stops human AGS gastric cancer cells after G1 phase (103). Papain hydrolysate purified from *Chlorella pyrenoidosa* showed anti-proliferation activity against HepG2 (104). Recently, heptapeptide (GPLGAGP) isolated from hydrolysates of *Enteromorpha prolifera* showed the most potent inhibitory activity against NCI-H460 lung cancer cells (106).

### 2.2 The role of fungi-derived protein and bioactive peptides in preventive and clinical nutrition

Fungi are suitable alternative sources of food for humans which have a low environmental footprint, and could be a solution for a sustainable future for our planet (109–113). Mycoprotein, which is a proteinaceous wholefood produced from the continuous fermentation of the filamentous fungus *Fusarium venenatum*, is the most popular example that is commercialized and sold in different countries (UK, USA, Belgium, Germany, Denmark, Ireland, France, the Netherlands, Switzerland, Sweden etc.) as an ingredient in products marketed under the brand name Quorn™ (114). Mycoprotein product contains high-quality protein (11.5%), fiber (6%) composed of glucan-chitin matrix, sugar (0.8%), fat (2.9%), and minerals, such as selenium (20%), zinc (less bioavailable than meat), iron (lower than meat), manganese, calcium, and phosphorus, and a source of vitamin B2 (115). Also, it is devoid of trans-fats and cholesterol. Due to its healthy nutritional profile, the consumption of mycoprotein is increasing worldwide (112). For example, including mycoprotein in the daily diet helps maintaining glycaemic control (116). Furthermore, edible mushrooms have tremendous health benefits including anti-inflammatory, antioxidant, anti-cancer, anti-diabetic and anti-obesity properties (117, 118).

#### 2.2.1 The role of fungi-derived protein and bioactive peptides in weight management

Owing to its sufficient amount of fiber and high protein content, mycoprotein plays a vital role in fighting hunger, decreasing body weight, and reducing energy intake. Literature have shown that mycoprotein ingestion reduces energy intake and increases satiety effects in obese and overweight individuals (119, 120). A study with 36 overweight and obese adults showed that 132 g energy-matched mycoprotein-based preload meal compared to a chicken meal for 180 min reduced energy intake by 10% (120). This could partly be due to the thermogenic and satiety effect of mycoprotein (121). Further longer-term studies are needed to investigate the potential of mycoprotein in the prevention of obesity and T2DM.

Interestingly, *Ganoderma lucidum*—a medicinal mushroom—showed anti-obesity activities by modulating the gut microbiota (decreased the ratio of Firmicutes to Bacteroidetes and levels of Proteobacteria) in high-fat diet (HFD)-fed mice, suggesting as a prebiotic to reduce weight (122). The finding also showed that supplementation of water extract of *Ganoderma lucidum* mycelium to HFD-fed mice daily for 2 months by oral gavage results in a dose-dependently decreased weight gain and both epididymal and subcutaneous fat accumulation, and reduced inflammation and insulin resistance compared with untreated controls. Given scarce studies in the area, extensive research and clinical studies demonstrating the safety and effectiveness of fungi-derived bioactive peptides are worthy of further investigation.

#### 2.2.2 The role of fungi-derived protein and bioactive peptides in improving cardio-metabolic health

Mycoprotein ingestion also advantageously modifies blood lipid profiles, as indicated by studies (123, 124). The greatest benefits have been observed in subjects with elevated cholesterol levels at baseline (124). A study conducted on 17 healthy participants indicated that ingestion of mycoprotein-containing products (191 g) per day for 3 weeks reduced total cholesterol, lowered low-density lipoprotein and improved high-density lipoprotein by 13, 9, and 12%, respectively (125). A randomized trial was conducted in a home setting among 72 overweight, hypercholesterolaemic adults showed that mycoprotein ingestion for 4 weeks reduces serum cholesterol, low-density lipoprotein level, blood glucose, and C-peptide concentrations by 6, 10, 13 and 27%, respectively (123). This implies that mycoprotein may improve peripheral insulin sensitivity, suggesting a role in preventive and clinical nutrition in reducing the risk of obesity and diabetes mellitus in adults. However, further investigation is warranted to consolidate this evidence. Consumption of edible mushrooms decreases plasma triglyceride, total cholesterol, and low-density lipoprotein (126). Moreover, bioactive peptides obtained from different edible mushroom species, such as *Pholiota adiposa, pleurotus cornucopiae, Hypsizygus marmoreus, Agaricus bisporus, Tricholoma giganteum*, *Ganoderma Lucidum*, and shiitake mushroom (*Lentinula edodes*) exhibited ACE inhibitory activities (127–133). A detailed summary of the beneficial effects of bioactive peptides has been provided in Table 1.

**Table 1:** A summary of the purported beneficial effects of algae, fungi and insect-derived peptides.

Source	Species	Title of the article with references	Year of publication	Name of bioactive peptide/amino acid sequence	*In vivo*/*In vitro*	Beneficial effects of the peptide	Future research areas
Algae	*Spirulina platensis/Arthrospira platensis*	Anti-obesity effects of *Spirulina platensis* protein hydrolysate by modulating brain-liver axis in high-fat diet fed mice (65)	2019	Spirulina platensis protein peptide hyrdolyste (SPPH)	*In vivo* animal (C57BL/6J mice)	Reduce body weight, serum glucose and total cholesterol	The molecular mechanism between Acadm and Acaca or Acsl1 warrants further study
		Purification and identification of anti-obesity peptides derived from *Spirulina platensis* (66)	2018	CANPHELPNK, NPVWKRK	*In vitro* using mouse 3T3-L1 preadipocytes and normal liver cells (L-O2)	Exhibit inhibitory effects on 3T3-L1preadipocyte proliferation, and reduce the accumulation of triglyceride	The detailed mechanism of action for the identified peptides needs to be explained
		Protein hydrolysate from *Spirulina platensis* prevents dexamethasone-induced muscle atrophy via Akt/Foxo3 signaling in C_2_C_12_ myotubes (59)	2022	Spirulina protein hydrolysate (SPH)	C_2_C_12_ cell line (CRL-1772)	Inhibits muscle atrophy mainly by activating the Akt/FoxO3a signaling pathway, increased myotube length and diameter in C_2_C_12_ cells,	To translate the effect of SPH as a healthy functional food material for the prevention, treatment, and improvement of muscle atrophy, *in vivo* study using animal models is required
		Identification of anti-diabetes peptides from *Spirulina platensis* (87)	2019	GVPMPN, RNPFVFAPTLLTVAAR and LRSELAAWSR	*In vitro*	Inhibition of α-amylase, α-glucosidase and DPP-IV	*In vivo* studies are important
		Characterization and antitumor activity of protein hydrolysates from *Arthrospira platensis (Spirulina platensis)* using two-step hydrolysis (107)	2016	AGGASLLLLR, KFLVLCLR(KR),LCLR (LR), LAGHVGVR	*In vitro* and *in vivo* (using nude mice)	Dose dependent inhibitory effect against breast cancer cells (MCF-7), lung cancer cells (A549), gastric cancer cells(SGC-7901), colon cancer cells (HT-29), liver cancer cells(HepG2)	Further study is warranted to divulge why the identified peptides did not inhibit cancer growth in a time dependent manner
		*In silico* and *in vitro* assessment of bioactive peptides from *Arthrospira platensis* phycobiliproteins for DPP-IV inhibitory activity, ACE inhibitory activity, and antioxidant activity (79)	2022	Phycobiliprotein hydrolysates (PBPHs)	*In silico* and *in vitro*	ACE inhibitory activity, DPP-IV inhibitory activity, antioxidant activity	*In vivo* study is required
	*Chlorella vulgaris*	Antihypertensive effects, molecular docking study, and isothermal titration calorimetry assay of angiotensin I-converting enzyme inhibitory peptides from *Chlorella vulgaris* (80)	2018	Val-His-Trp (VHW), Thr-Thr-Trp (TTW)	*In silico* and *in vivo* (SHR). Molecular docking and Isothermal titration calorimetry (ITC) assay	VHW reduces SBP from 234 to 184 mmHg happened at 2 h. TTW reduces DBP from 180 to 140 mmHg at 2 h. Thus, VHW and TTW could be used to treat different hypertensive phenotypes or cooperate together.	Enrichment of the tryptophan content of the identified peptides is important
			Anticancer and antioxidant activities of the peptide fraction from algae protein waste (103)	2010	VECYGPNRPQF	*In vitro*	The identified peptides effectively induced cell death and inhibited the growth of AGS cells after G1 phase. The antioxidant activity of the peptide fraction was about 26-fold stronger than that of Trolox.	*In vivo* study is warranted
	*Chlorella pyrenodosa*	Separation, antitumor activities, and encapsulation of polypeptide from *Chlorella pyrenoidosa* (104)	2013	*C. pyrenoidosa* antitumor polypeptide (CPAP)	*In vitro*	Anti-proliferative activity against human liver cancer HepG2 cells.	Detailed encapsulation mechanisms, and *in vivo* study is required. Amino acid sequence of the peptide was not determined
	*Enteromorphia Prolifera*	Preparation and Characterization of an Anticancer Peptide from Oriental Tonic Food *Enteromorpha prolifera* (106)	2022	HTDT-6-2-3-2	*In silico*, *in vitro* and molecular docking	Inhibitory activity against NCI-H460 human cancer cell lines	HTDT-6-2-3-2 may also be novel inhibitors for ACE and DPP4. Future experiments will be conducted to verify this assumption
Fungi	*Pholiota adipose kumm* (Yellow-cap fungus)	Production and characterization of antihypertensive angiotensin I-converting enzyme inhibitor from *Pholiota adipose* (127)	2006	GEGGP	*In vivo* (SHR)	ACE inhibitory activity (decrease SBP by 22 mmHg)	ACE inhibitory activity is weaker than captopril. Research may require to enhance the ACE inhibitory activity of the identified peptide
	*Hypsizygus marmoreus*	Characterization of an antihypertensive angiotensin I-converting enzyme inhibitory peptide from the edible mushroom *Hypsizygus marmoreus* (129)	2013	LSMGSASLSP	*In vivo* (SHR)	ACE inhibitory activity (decrease SBP by 26 mmHg)	Human study is required for translating in to the actual health benefits.
	*Pleurotus cornucopiae*	Characterisation of a new antihypertensive angiotensin I-converting enzyme inhibitory peptide from *Pleurotus cornucopiae* (128)	2011	RLPSEFDLSAFLRA, RLSGQTIEVTSEYLFRH	*In vivo* (SHR)	ACE inhibitory activity (decrease SBP by 50 mmHg)	Further studies are necessary to reduce the molecular weight of the identified peptide for application into the medicinal industry.
	*Tricholoma giganteum* (Giant mushroom)	Isolation and characterization of a novel angiotensin I-converting enzyme inhibitory peptide derived from the edible mushroom *Tricholoma giganteum* (131)	2004	GEP	*In vivo* (SHR)	ACE inhibitory activity (decrease SBP by 36 mmHg)	Human study is required for translating in to the actual health benefits.
	*Agaricus bisporus* (button mushroom)	Novel angiotensin I-converting enzyme inhibitory peptides derived from edible mushroom *Agaricus bisporu*s (J.E. Lange) Imbach identified by LC–MS/MS (130)	2014	AHEPVK, RIGLF, PSSNK	*In vitro*	ACE inhibitory activity	*In vivo* study is required
	*Ganoderma Lucidum*	Isolation and characterization of three antihypertension peptides from the mycelia of *Ganoderma lucidum* (Agaricomycetes) (132)	2019	QDVL, QDVL, QLDL	*In vitro*	ACE inhibitory activity	Future studies on the cellular mechanism by which the identified peptides inhibit hypertension should be conducted to confirm this finding
Insects	*Tenbrio Molitor* (Yellow mealworm)	Identification and *in silico* analysis of antithrombotic peptides from the enzymatic hydrolysates of *Tenebrio molitor* larvae (165)	2019	SLVDAIGMGP, AGFAGDDAPR	*In silico* and molecular docking	Antithrombotic effect	Before applying it to the clinic, it still needs to prove the antithrombotic effect *in vivo* through a large number of experiments.
	*Tenbrio Molitor* (Yellow mealworm), Cricket (*Gryllodes sigillatus*), Locust (*Schistocerca gregaria*)	Evaluation of ACE, α-glucosidase, and lipase inhibitory activities of peptides obtained by *in vitro* digestion of selected species of edible insects (162)	2020	KVEGDLK, YETGNGIK, AIGVGAIR, IIAPPER, FDPFPK	*In vitro*	Inhibition of ACE, pancreatic lipase and α-glucosidase	Mechanism of lipase inhibition by peptides is still poorly understood, requiring further study
	Silkworm(*Bombyx mori*)	Novel tripeptides with α-glucosidase inhibitory activity isolated from silk cocoon hydrolysate (163)	2011	E5K6	*In vitro*	α-glucosidase inhibitory activity	Further studies are needed on the side effect of the identified peptide using animals
		A novel angiotensin-І converting enzyme (ACE) inhibitory peptide from gastrointestinal protease hydrolysate of silkworm pupa (*Bombyx mori*) protein: biochemical characterization and molecular docking study (148)	2015	Silkworm pupa protein hydrolysate (SPPH)/Ala-Ser-Leu (ASL)	*In vitro*, molecular docking	Competitive ACE inhibitor	*In vivo* study is required

#### 2.2.3 The role of fungi-derived protein and bioactive peptides in muscle anabolism

Furthermore, mycoprotein ingestion (121, 123, 134–137) increases muscle protein synthesis rate when taken as a whole food, an isolated source or in a blended form, particularly in young individuals. Mycoprotein (at a dose of 60–80 g) ingestion led to slower but more sustained hyperaminoacidemia and hyperinsulinemia when compared with 20 g protein-match milk (121). Similarly, mycoprotein ingestion (70 g) resulted in a less rapid but more sustained increase in serum insulin levels, peaking at 30 min after consumption when compared with milk protein (136). However, a large amount of mycoprotein ingestion coupled with its satiating properties (138, 139) may not be a pragmatic approach in compromised individuals (anorexic older adults, patients with sarcopenia and cachexia). In such situations, protein concentrate from mycoprotein, and BCAA-enriched mycoprotein may be a feasible option, but further investigation is warranted.

### 2.3 The role of insect-derived protein and bioactive peptides in preventive and clinical nutrition

Given that more than 2,100 edible insects are found worldwide, their nutritional composition is difficult to generalize (140). However, many edible insects contain high-quality protein (141), unsaturated fatty acids (linolenic and linoleic acid), micronutrients (iron, zinc, calcium, potassium, magnesium, manganese, copper, vitamin E, vitamin K, vitamin B_2_, and vitamin B_12_) and fiber (142–145). Interestingly, iron and zinc found in crickets, grasshoppers and mealworms have been shown highly bioavailable compared to sirloin beef (143). Currently, common edible insect species are house cricket (*Acheta domesticus*), African palm weevil (*Rhynchophorus phoenicis*), yellow mealworm (*Tenebrio molitor*), mopane worm (*Gonimbrasia belina*), domesticated silkworm (*Bombyx mori*),and honeybee (*Apis mellifera*) (146). Edible insects are a sustainable source of protein for food and feed given their lower water and land consumption, high feed conversion efficiency, low greenhouse gas emissions compared to livestock, short life cycle, and rapid intrinsic growth rate (147, 148).

#### 2.3.1 The role of insect-derived protein in muscle protein synthesis: insight for sarcopenia prevention

Studies showed that ingestion of lesser mealworm-derived protein (149), and cricket protein powder (150) were capable of stimulating MPS at rest and after exercise in young men. In particular, similar postprandial amino acid availability and MPS rates occurred after ingestion of 30 g lesser mealworm-derived protein to an equivalent amount of milk protein concentrate (149). In addition, no significant difference was observed in the mammalian target of rapamycin (mTORC1) signaling between cricket, pea, and whey protein despite a higher concentration of EAA and leucine occurring after ingestion of whey (150). Similarly, the EAA, BCAA, and leucine plasma levels peaked earlier in soy, whey and beef-derived protein than for cricket and lesser-mealworm (151, 152). The observed differences may be attributed to the slowly digested properties of lesser mealworm and cricket-derived compared to animal-derived proteins.

The high muscle protein anabolic potential of insect-derived proteins provides a strong initiative for integrating insects into the diet, especially in Western countries where acceptance of insect consumption is low. The high quality of insect-derived proteins coupled with their high amounts of micronutrients and antioxidants (153–155), will be of particular relevance in populations that consume less protein and suffer from anabolic resistance (156), such as the older and more clinically compromised populations.

#### 2.3.2 The role of insect-derived bioactive peptides in preventing obesity, diabetes and hypertension

Although human studies are still lacking, peptides and bioactive compounds derived from edible insects could provide health benefits, such as anti-oxidant (157), anti-obesity (158), anti-diabetic (159) and anti-hypertensive properties (160). A study showed that yellow mealworm larvae powder administration in obese mice reduced body weight gain by decreasing lipid accumulation and triglyceride content in adipocytes (158), thus demonstrating the potential to induce weight loss. Moreover, an ethanol extract of the Korean horn beetle *Allomyrina dichotoma* injection into the brain tissue of obese mice reduced both endoplasmic reticulum (ER) stress and hormone-induced change in feeding behaviour (159). Thus, it could imply in preventing and treating obesity and T2DM since reduction of ER stress enhances insulin-producing beta cells (161).

Antioxidant activity in edible insect species was reported, including free radical-scavenging activity, and iron-chelating ability (157). Moreover, inhibition of ACE, pancreatic lipase and α-glucosidase have been observed in peptides derived from yellow mealworm(*Tenbrio Molitor*), silkworm (*Bombyx mori*), cricket (*Gryllodes sigillatus*) and locust (*Schistocerca gregaria*) (160, 162–164). Besides the ACE inhibitory activity, peptides derived from *Tenebrio molitor* larvae showed strong antithrombotic properties (165). However, the evidence mentioned above (157–160, 162–165) requires further human studies to translate into actual health benefits.

### 2.4 Food safety of edible insects

Apart from the nutritional, environmental and health benefits of edible insects, food safety issues need to be considered. This includes anti-nutrient content, allergenic potential, microbial safety and chemical contamination. Anti-nutrients, such as phytic acid, tannin, saponins, oxalate and cyanogenic glycosides have been determined in edible insects (145, 166, 167). More research on the anti-nutritional properties is necessary. Allergic reactions that could be triggered by insect consumption include nausea, vomiting, diarrhea, asthma, and skin reactions (168, 169). Commonly, tropomyosin and arginine kinase pan-allergens have been identified (168–170). Cross-reactivity and co-sensitisation between (insects and house dust mites) and (insects and crustaceans) occur because of these pan-allergens (168, 169). Therefore, clear labelling and communication of the allergenic potential of insects to the consumer is required since insect protein allergenicity is not removed by thermal treatments (168).

Concerning microbial safety, different microbiota have been found on raw edible insects, including lactic acid bacteria, Enterobacteriaceae, fungi, mesophilic aerobes, spore-forming bacteria, and foodborne pathogens (171), albeit no outbreaks associated with pathogens have been reported in the scientific literature. Thus, effective decontamination, and good hygiene practices during rearing, processing and storage are essential to minimize the microbial hazards of insect-containing food products (172).

Regarding chemical contamination of edible insects, low levels of contaminants (the level below the legal maximum amount), such as dioxin compounds, pesticide residue, arsenic, cadmium, copper, nickel, mercury, lead, and tin have been identified (173, 174). Insect species, rearing conditions, feed substrate including the packaging material of the substrate, and post-harvest processing were factors for the chemical contamination of edible insects (172, 175). Regular monitoring of the rearing environment, strong supplier auditing and certification process could reduce the risk of heavy metal contamination (176). Additionally, using naturally derived biopesticides from plants, micro-organisms or beneficial insects, such as neem oil, *bacillus thuringiensis*, and insect pheromones could decrease the deposition of residual pesticides (177). Future research should focus on determining the best processing conditions to create insect protein isolates with good functional characteristics, cost-effectiveness, and environmental sustainability that can be used in food formulation.

### 2.5 Challenges and strategies to consume edible insects

Despite a surfeit of health benefits, and the potential of edible insects to represent an environmentally sustainable nutrient source, willingness to consume insects as a food is low in Western countries (178, 179); albeit they aren’t against advances and food innovations. Food disgust (the product goes beyond the internalized norm of what food is), lack of familiarity with insect consumption, neophobia (hesitance to consume unfamiliar food), lack of product information, curiosity or sensation seeking and food technology neophobia were the identified drivers for low acceptance of insect consumption (178–180). As a result, different strategies have been suggested to convince consumers, such as underscoring that insects are nutritious, harvesting properly on controlled farms, making insect products delicious (enhancing the culinary experience), familiarizing insect-based products, integrating them in unrecognizable forms in familiar products, marketing insect-based products by taste, using celebrities to promote the product, targeting specific groups (sensation seekers, adventurous consumers or children), information provision from a nutritional and environmental standpoint, devising market approaches (stylistic images, choosing supermarkets for retailing, and using promotional tools such as buy-one-get-one-free, and discounts) (176, 181).

## 3 Conclusion

In conclusion, proteins derived from algae, fungi, and edible insects are high-quality proteins as animal sources and demonstrate significant potential as a sustainable source of bioactive peptides, which are metabolically potent and have negligible adverse effects. They show promise to prevent and treat diseases associated with oxidative stress, obesity, diabetes, cancer, cardiovascular disease (especially hypertension), and neurodegenerative diseases. Given the abundance of algae, fungi and insect peptides performed *in vitro* or *in vivo* animals, further research, validation and clinical studies are needed to fully establish their safety, efficacy and practical application in preventive and clinical nutrition. Additionally, social and behavioral change communication strategies would be important to increase health awareness of nutritional benefits and promote consumer acceptance of alternative protein sources.
